# Study of the B-Dot Sensor for Aircraft Surface Current Measurement

**DOI:** 10.3390/s22197499

**Published:** 2022-10-03

**Authors:** Chen Tong, Zemin Duan, Yeyuan Huang, Shanliang Qiu, Xiaoliang Si, Zhibao Li, Zhijie Yuan

**Affiliations:** 1 School of Electrical Engineering and Automation, Hefei University of Technology, Hefei 230009, China; 2 Anhui Provincial Key Laboratory of Aircraft Lightning Protection, Hefei 230009, China; 3 Aerospace Science and Technology Key Laboratory of Strong Electromagnetic Environment Protection Technology, Hefei 230009, China; 4 School of Mathematics, Hefei University of Technology, Hefei 230009, China

**Keywords:** B-dot, calibration, magnetic field measurement, lightning current measurement

## Abstract

The B-dot sensor is a type of Rogowski coil widely used in the measurement of current. However, the accuracy of the B-dot for measuring aircraft high-frequency lightning current is greatly affected by factors such as numerical integration drift, high-frequency oscillation, and calibration. In this study, a new design and optimization for improving the B-dot measuring accuracy was carried out. To correct the drift of the numerical integral of the measurement signal in differential mode, the measuring current was reconstructed based on the nonlinear least squares method. The sensor was then optimized by isolating the sampling resistance and matching the impedance with a voltage follower. A low-cost coaxial loop calibration system was also designed to calibrate the high frequency and strong magnetic fields more accurately. Finally, the optimized B-dot sensor accuracy was greatly improved with a measuring range of 30 kA/m, an error of 3.1%, and a high-frequency response of 50 MHz. Our study greatly increases the accuracy of measuring aircraft high-frequency lightning current.

## 1. Introduction

Aircraft are easily struck by lightning in the upper air or on the ground, thus threatening the safety of flights. To ensure the safety of aircraft, mandatory rules and regulations have been stipulated by the airworthiness authorities of various countries since the 1930s. The lightning test is the key step for determining the lightning airworthiness of an aircraft. In this step, the measurements of lightning current, for example, the current distribution and the magnetic field, are an important part of airworthiness conformity certification [1,2,3]. Measuring surface current and magnetic field distribution is necessary not only in the research field of aircraft lightning protection, but also in many other fields, such as electromagnetic railguns, high-power microwave, fault diagnosis [4,5,6], etc. Therefore, it is essential to study the sensors.

There are several sensors that have been developed for measuring aircraft lightning test current, such as giant magnetoresistance (GMR), tunnel magnetoresistance (TMR), and B-dot. These three sensors are widely used for measuring transient magnetic fields. Huang et al. designed a measurement system based on a GMR sensor and verified the feasibility of this system [7]. To measure the three-dimensional transient magnetic field component generated by a discharge current, Cheng et al. designed a 3-D sensor based on a TMR chip, which was successfully applied in monitoring the overhead catenary of a high-speed railway lightning strike [8]. Although these systems based on GMR or TMR have many advantages, they are insufficient to measure higher frequency current, especially the magnetic field generated by the current component H in the aircraft lightning test [9]. Additionally, the costs of these systems are very high.

The B-dot sensor is a non-closed-loop Rogowski coil [10] with good performance in measuring high-frequency current, and its cost is very low. Due to this superior performance and low cost, B-dot is widely used in the measurement of lightning current, partial discharge pulse current, magnetic insulation transmission line [11,12], and other pulse current measurement fields. B-dot is also widely used in measuring the electromagnetic field of lightning. In 2003, Sebo et al. used the coil B-dot sensor to measure the magnetic field inside and outside an aircraft for the lightning test. By injecting a 15 kA pulse current with a front time at the microsecond level, the influence of different cover materials on the internal magnetic field was investigated [13,14]. Other researchers have also designed lightning current measurement systems based on B-dot, usually installed on airplanes or communication towers, and analyzed the sensitivity of the sensors [15,16].

The B-dot’s main design challenge was to improve its accuracy. The selection and matching of load resistance, trend term removal, and a calibration system with high amplitude are the main factors affecting accuracy. Constructing an accurate equivalent circuit is the premise of choosing load resistance. The circuit model of B-dot can adopt the equivalent loop of the Rogowski coil [17,18,19,20,21,22]. Kut assumed that the input resistance and the input capacitance of the measurement system were 1 MΩ and 5 pF, and determined the selection method of the winding number according to the symmetry of the equivalent circuit and phase frequency response curve [23,24]. Bogdan Dziadak compared the difference between 8/20 μs and 4/16 μs waveforms measured by B-dot sensors in different winding configurations and sizes [25]. According to the calculation formula of stray parameters of the coil [26,27,28], the transfer function of the system can be derived. The improper selection of load resistance will lead to oscillations when measuring high-frequency signals [29]. This is because the poles of the transfer function of the sensor system are imaginary and too close to the virtual axis. In addition, the B-dot sensor requires an integral operation to restore the magnetic field and current, which is another source of error. Integration can be achieved by analog integration and numerical integration. The structure of analog integrators is simple and easy to realize, but the main disadvantages of analog integrators are fixed time constant, saturation, and drift, and these disadvantages limit their use. Numerical integration can compensate for these shortcomings, but there is a serious integral drift problem in numerical integration, which leads to inaccurate or even impossible recognition of the rear edge of the waveform. B-dot post-processing is similar to D-dot. Jakubowski et al. reconstructed an electric field time-domain waveform through numerical integration by using a digital processing algorithm [30]. Yao Lijun et al. used data with an amplitude of 0 to remove the bias of D-dot measurement data, but did not consider the accuracy of half-peak time identification [31]. Jóśko et al. removed the trend term by independent signal segmentation and integration for each sub-region [32]. As lightning current is a broadband signal, this method will lead to a large number of sub-intervals and a large amount of sampled data. Wei Bing et al. used a low-pass filter with a negative time constant to suppress the trend term to a certain extent [33]. The current commonly used de-trending algorithm cannot completely remove the trend term, which leads to a gradual increase in error over time, and the deviation of the back edge of the measured waveform. The accuracy of the calibration system is a guarantee for B-dot accuracy. Institute of Electrical and Electronics Engineers(IEEE) standards provide the sensor measurement principle and the calibration mode in the field of AC overhead power lines [34]. Calibration can be performed in the frequency domain or the time domain by a Transverse Electromagnetic Wave(TEM) cell [35]. Zhang Guangwei et al. studied the frequency domain characteristics of the magnetic field waveform generated by the switch through a wavelet transform [36]. Hongzhi Ouyang et al. proposed an improved Al-Alaoui algorithm and used a single coil for calibration. The traditional calibration method does not take into account the non-uniformity of the magnetic field in the induction area, so there is a certain error between the theoretical formula and the practice, and the calibration equipment that generates high intensity and a high-frequency magnetic field is often expensive.

To improve the B-dot sensor accuracy, this paper carried out study on current waveform reconstruction, sampling resistance selection, and calibration system construction based on [27,28,37]. This study is organized as follows: the first section mainly describes the principle and equivalent model of the B-dot sensor; the second section studies the selection range of load resistance and the method of current waveform reconstruction, providing an evaluation method of the inducted voltage of the coaxial loop calibration system considering non-uniformity; the third section analyzes the device selection and trial manufacture of the post-stage divider isolation circuit, and calibrates the optimized B-dot sensor; and the last section summarizes this study.

## 2. The Basic Theory

### 2.1. B-Dot Measurement Principle

**Figure 1:**
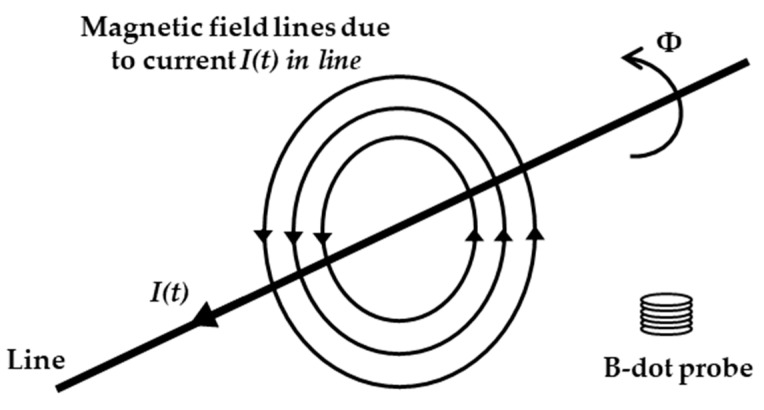
The measurement principle of the B-dot sensor. The current creates a magnetic field and induces an electromotive force in the coil.

The B-dot sensor can measure the current indirectly by measuring the magnetic field directly. Figure 1 presents the B-dot sensor measuring principle. The current that flows in the long straight wire is *I(t)*, and Φ denotes the magnetic flux that caused by *I(t)*. The changing current results in a changing magnetic field, and the alternating magnetic field, furthermore, brings in an induced electromotive force in the coil.

According to Faraday’s law, the induced voltage of the coil can be expressed as:

(1) $$V=N\oint \vec{E}\times \hat{d}l=-\frac{\partial }{\partial t}\left(NBA\right),$$

where *N* is the number of turns of the coil. From Equation (1), it can be seen that the induced voltage of the coil is linearly related to the rate of change in the magnetic field strength, and the magnetic field strength *H* is

(2) $$H=\frac{1\times \int Vdt}{\mu_{0}\mu_{r}NA}.$$

According to Equation (2), the output proportional coefficient *K_H_* of the sensor could be given as

(3) $$K_{H}=\frac{H}{\int Vdt}=\frac{1}{\mu_{0}\mu_{r}NA}.$$

The unit of *K_H_* is A/m/V/s, where $\mu_{0}$ is the vacuum permeability $\mu_{0}$ = 4π*10^−7^ H/m. $\mu_{r}$ is the relative permeability of the material in the sensing area of the coil. According to the continuity principle on the interface,

(4) $$H_{1t}-H_{2t}=J_{l}.$$

The external tangential magnetic field is equal to the linear density of the conducting current on the interface when the internal tangential magnetic field is close to 0. Therefore, the linear density of the conducted current can be then obtained by measuring the magnetic field.

### 2.2. Lumped Parameter Model

**Figure 2:**
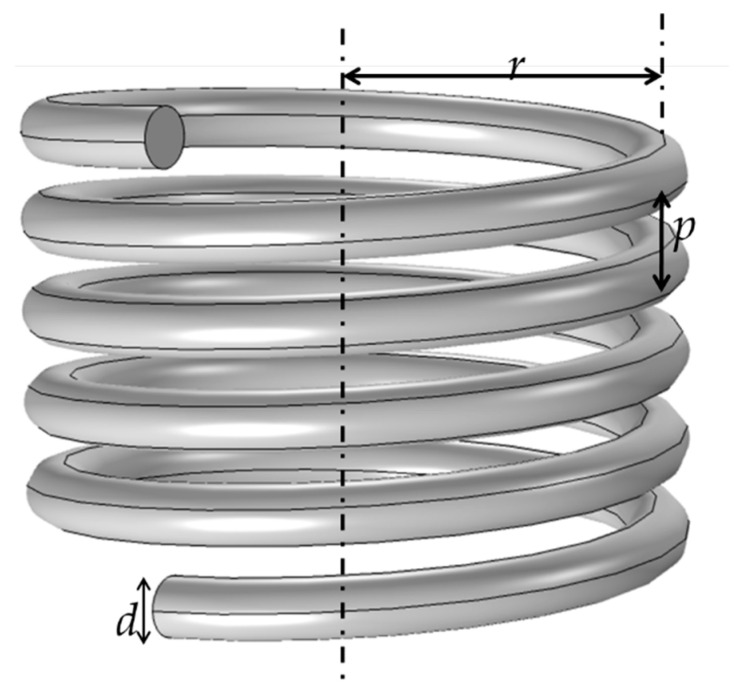
The coil dimensions including the radius of the solenoid (*r*), the diameter of the enameled wire (*d*), and the wire spacing (*p*).

In order to investigate the frequency band performance of the sensor, it is necessary to extract the stray parameters of the coil to thus determine the equivalent circuit. The size diagram of the coil is shown in Figure 2, where *r* is the radius of the solenoid (mm), *d* represents the diameter of the enameled wire (mm), *n* denotes the number of coils wound, and *p* is the wire spacing.

**Figure 3:**
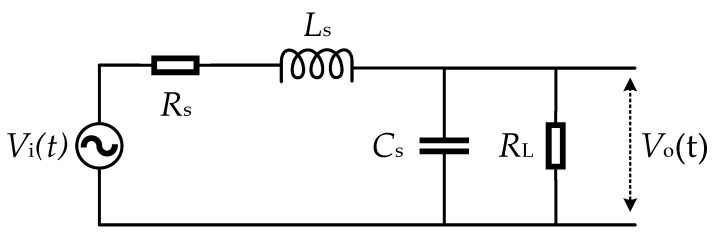
The equivalent circuit.

The equivalent circuit of the sensor is shown in Figure 3, where *R*_S_, *L*_S_, and *C*_S_ are the equivalent resistance, inductance, and capacitance of the coil, respectively, and *R*_L_ represents the load sampling resistance.

The *R*_S_ can be calculated by

(5) $$R_{S}=\frac{\rho L}{S}=\frac{8\rho rN}{d^{2}},$$

where ρ is the resistivity of the enameled wire, the resistivity of copper is 1.75 × 10^−5^ Ω·mm, L represents the length of the wire, S stands for the cross-sectional area of the wire; *r* denotes the radius of the solenoid, and d is the diameter of the enameled wire. The *L*_S_ can be calculated by

(6) $$L_{S}\approx \frac{4\pi^{2}r^{2}N^{2}\times 10^{-6}}{9r+10Nd+8.4d+3.2(Nd^{2}/r)}.$$

The equation for calculating the stray capacitance of the coil is

$$C_{\mathrm{tt}}=\frac{2\varepsilon_{0}\pi (2r+d)}{\sqrt{{\left(\frac{p}{d}\right)}^{2}-1}}\times \arctan\sqrt{1+\frac{2}{\frac{p}{d}-1}}$$

(7) $$C_{S}=\frac{C_{\mathrm{tt}}}{N-1},$$

where *C*_tt_ is the inter-turn capacitance and C_S_ represents the stray capacitance, and the vacuum dielectric constant is 8.854 × 10^−15^ F/mm. 

## 3. The Optimized Design of the Sensors

The dynamic range and accuracy of the B-dot sensor are optimized. At present, most coil-type B-dot designs are directly connected to an oscilloscope through coaxial cables or optical fibers without a voltage division circuit, which limits the measurement range. In addition, the stray capacitance and inductance of the coil and measurement system will also result in narrow signals on the rising edge of the measurement. Another issue is the resonant frequency *f*_0_, which is determined by Ls and Cs. Since the system, composed of the sensor and the measurement module, has a pair of conjugated complex poles, this means that the state of the circuit is an underdamped state. When the frequency band of the measurement signal includes the resonant frequency, the waveform will superimpose the oscillation. The drift caused by numerical integration will increase the measurement error.

### 3.1. The Selection of the R_L_

According to the stability criterion of the system, the oscillation of the waveform by the sensor is caused by the conjugated complex poles because the pole is very close to the imaginary axis and the attenuation is very slow, which results in a reduction in accuracy, although the system is stable in theory. To avoid poor accuracy, it is necessary for the poles of the system to be optimized.

If the poles move away from the imaginary axis or become two identical negative real numbers, the oscillation could quickly attenuate or the oscillation could even be eliminated. The Laplace transfer function of the circuit presented in Figure 3 is

(8) $$H(s)=\frac{v_{o}(s)}{v_{i}(s)}=\frac{\omega_{0}^{2}}{s^{2}+\alpha s+\omega_{0}^{2}\beta }$$

where $\omega_{0}=\frac{1}{\sqrt{L_{s}C_{s}}}$, $\alpha =\frac{r_{s}}{L_{s}}+\frac{1}{C_{s}R_{L}}$, $\beta =\frac{r_{s}}{R_{L}}+1$.

For the output to satisfy the overdamping condition, the system needs to have two negative real poles. Therefore, it is necessary for α and β to meet

(9) $$\frac{\alpha^{2}}{4}-\omega_{0}^{2}\beta >0.$$

In addition, from the perspective of the speed of the system response, $\frac{L_{S}}{R_{L}}$ determines the response speed of the system. In order to enable the coil to reflect the real magnetic field waveform, it needs to meet the following:

(10) $$\frac{L_{S}}{R_{L}}<T_{r}.$$

Connecting inequalities (9) and (10), and substituting α, β, and ω_0_ in, the inequalities after simplification are

(11) $$\left\{ \begin{array}{l} f(R_{L})=aR_{L}{}^{2}+bR_{L}+c^{2}>0\\ R_{L}>\frac{L_{S}}{T_{r}} \end{array}\right..$$

In Equation (11), $a=R_{S}{}^{2}C_{S}{}^{2}-4L_{S}C_{S}<0$, $b=-2R_{S}L_{S}C$, $c=L^{2}$, and $\Delta =b^{2}-4ac$. The stray parameters of the coil are in the order of pF and *μ*H, so the first inequality is a quadratic function.

**Figure 4:**
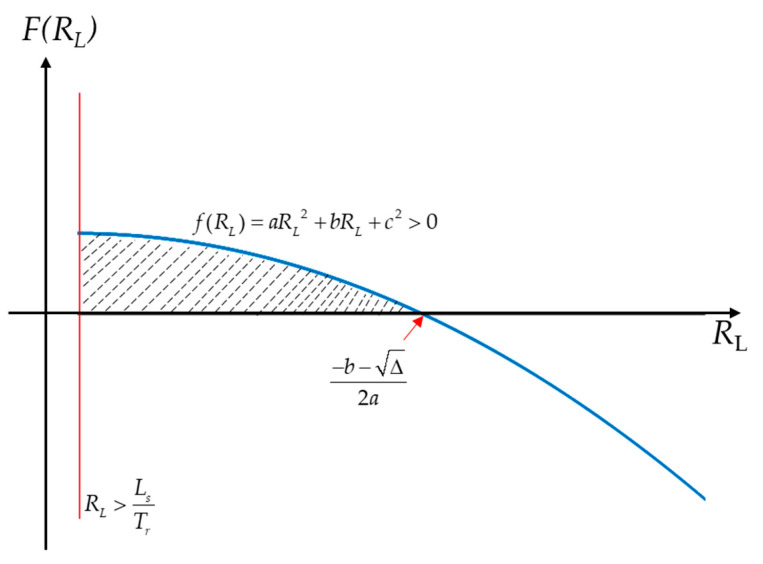
The deterritorialization of inequality systems. According to Equation (11), the value range of R_L_ is within the dotted line. The red and blue lines correspond to the two constraints of Equation (11), respectively.

From Figure 4, it can be seen that the solution condition of the inequality group should meet:

(12) $$\frac{-b-\sqrt{\Delta }}{2a}>\frac{L_{S}}{T_{r}}.$$

Thereby,

(13) $$L_{S}<\frac{R_{S}{}^{2}C_{S}{}^{2}-2R_{S}C_{S}T_{r}+T_{r}{}^{2}}{4C_{S}}.$$

The value range of the load resistance is $\frac{L_{S}}{T_{r}}<R_{L}<\frac{-b-\sqrt{\Delta }}{2a}$. In order to avoid the influence of the skin effect at high frequency, a larger load resistance value should be carefully selected, which allows for the possibility that the voltage divider ratio caused by the change in the coil resistance can be ignored.

### 3.2. Calibration Method

B-dot sensors need to be calibrated to verify linearity and to obtain output proportionality. The Helmholtz coil, single wire, and TEM cell, etc., are the most widely used calibration methods. However, calibration equipment with good performance is usually very expensive. A low-cost calibration source that retains a good performance is thus necessary.

**Figure 5:**
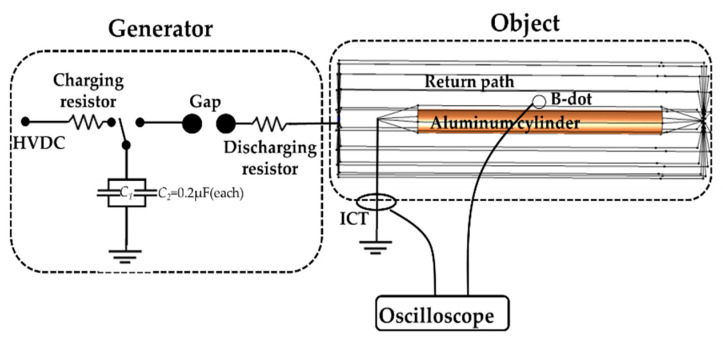
The B-dot calibration environment. The calibration system consists of a generator and object. The generator is connected in parallel by two capacitors with a capacitance value of 0.2 μF, and the high-voltage DC power charges the capacitors through a charging resistor. The coaxial loop generates a uniform magnetic field. The B-dot is placed in the middle of the aluminum barrel. A current sensor is used to collect the output current.

Figure 5 presents a calibration coaxial loop, with the calibration environment also illustrated, which includes a pulse current-generating device and a coaxial loop composed of an aluminum cylinder and wires. To ensure the uniformity of the current distribution, the number and length of the wires should be carefully selected. The calculation method for the coil-induced voltage of the environment in Figure 5 is analyzed below.

**Figure 6:**
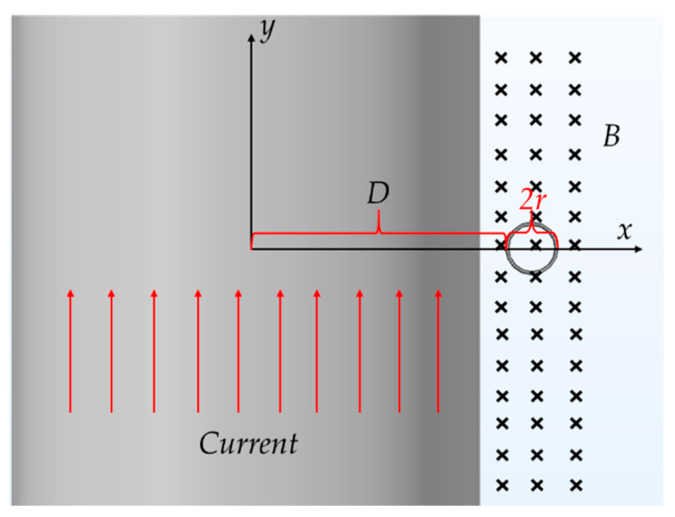
Induced voltage calculation in calibration environment. The current flows along the aluminum barrel, and the resulting magnetic field points in-plane along the tangent of the aluminum barrel, inducing an electromotive force in the B-dot.

In Figure 6, assume the distance from the coil to the center of the aluminum barrel is D. According to Equation (1), the induced voltage in the coil is

(14) $$\begin{array}{l} V=N\oint \vec{E}\cdot \hat{d}l=-\frac{N\mu_{0}\mu_{r}}{2\pi }\frac{\partial I}{\partial t}\iint_{}\frac{1}{x}dxdy,x,y\in {\left(x-r-D\right)}^{2}+y^{2}\leq r^{2}\\ =-\frac{N\mu_{0}\mu_{r}}{\pi }\frac{\partial I}{\partial t}\int_{0}^{r}\ln\left(\left(\sqrt{r^{2}-y^{2}}\right)+r+D\right)-\ln\left(r+D-\left(\sqrt{r^{2}-y^{2}}\right)\right)dy \end{array}.$$

A simplified form is

(15) $$V=-N\mu_{0}\mu_{r}\left(r+D-\sqrt{D^{2}+2Dr}\right)2\pi (D+r)\frac{\partial H}{\partial t}.$$

Generally, the voltage noise is approximately 1 mV. To obtain a higher signal-to-noise ratio, the output voltage of the coil needs to be larger than 10 mV and smaller than the saturation voltage of the operational amplifier chip. The measurement range is thus expanded by resistance division. In order to ensure an adequate signal-to-noise ratio and the stable operation of the rear circuit, the number of turns of the sensor needs to meet:

(16) $$V_{Min}\leq N\mu_{0}\mu_{r}\left(r+D-\sqrt{D^{2}+2Dr}\right)2\pi (D+r)\frac{R_{x}}{R_{L}}\frac{\partial H}{\partial t}\leq V_{Max},$$

where $\frac{\partial H}{\partial t}$ is the rate of change in the magnetic field, and $R_{x}$ is the sampling resistance of the x-segment range. *V_Min_* and *V_Max_* represent the lower limit and upper limit of the coil-induced voltage, respectively.

### 3.3. Reconstruction of the Signal

**Figure 7:**
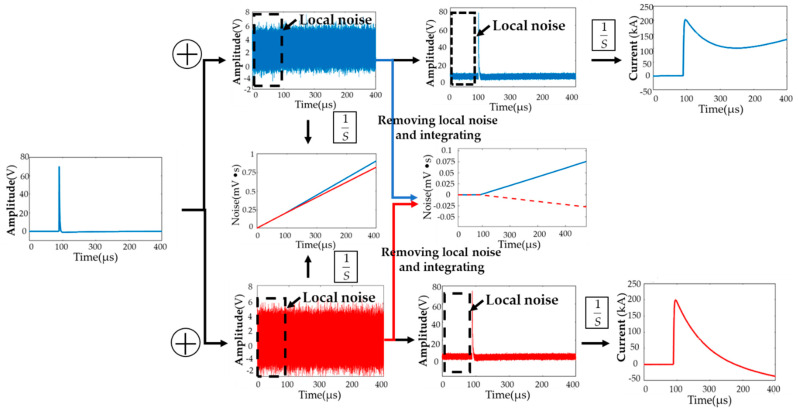
The integrated result of the original signal with different noise signals added after filtering out the local mean shows random updrift and droop effects.

Because the output of the coil is the differential of the magnetic field, it needs to be integrated to restructure the signal. The active and passive integrator of the analog integration can restructure the B-dot measurement signal, but the shortcomings of the analog integrator mainly include fixed time constant, saturation, and drift, and the measurement signal will deteriorate if the measurement system contains an optoelectronic circuit. These disadvantages limit the use of analog integrators. Fortunately, numerical integration can compensate for these shortcomings, but there is a serious integration drift problem in numerical integration, as shown in Figure 7.

As shown in Figure 7, the clear signals are superimposed with different noises. After the signal without local noise is integrated, updrift (blue line) and droop (red line) effects appear. This waveform distortion leads to a failure to identify the half-peak time. The droop effect usually causes designers to think that the bandwidth is insufficient, but this droop effect is caused by numerical integral drift. In Figure 7, after removing the same local mean of the noise signal and integrating it, it can be seen that the global mean of the up noise (blue line) is larger than the local mean, whereas the global mean of the down noise (red line) is smaller than the local mean. As can be seen in Figure 7, this slight difference will also lead to a severe distortion of the waveform after integration. The reason why the wavefront time distortion is smaller than the half-peak time is that the error of this numerical integration is cumulative. 

In order to avoid waveform distortion, the current waveform is reconstructed by using the nonlinear least squares method. The waveform after noise integration is approximately a straight line, and the measured current formula can be constructed as

(17) $$\hat{I}(t)=I_{0}\left(e^{-\alpha t}-e^{-\beta t}\right){\left(1-e^{-\gamma t}\right)}^{2}+at+b,$$

where *at* + *b* represents the term of noise after integrating, and $I_{0}$, α, β, and γ are the parameters of the current waveform. The estimated values of these parameters can be obtained using the nonlinear least squares method. The reconstructed current waveform can then be obtained as

(18) $$\hat{I}(t)={\hat{I}}_{0}\left(e^{-\hat{\alpha }t}-e^{-\hat{\beta }t}\right){\left(1-e^{-\hat{\gamma }t}\right)}^{2},$$

where ${\hat{I}}_{0}$, $\hat{\alpha }$, $\hat{\beta }$, and $\hat{\gamma }$ are the estimated values of $I_{0}$, α, β, and γ, respectively.

## 4. Simulation and Experiment

### 4.1. B-Dot and Circuit Design

In this study, a surface current sensor is designed to verify the proposed method. The diameter of the enameled wire is 0.1 mm, and the radius of the winding post is 17 mm. Assuming the lower measuring limit of the magnetic field is 1 A/m, then Equation (17) should meet $\frac{\partial H_{compA}}{\partial t}=0.7\times 10^{6}$. The output voltage of the single-turn coil is thus 0.685 mV. According to Equation (17), the number of coil turns is determined to be 10. The value of R_L_ ranges from 696.3 Ω to 2.8 kΩ (take R_L_ = 2.4 kΩ). The OPA659 chip, with a high bandwidth, unity gain stability, low drift, and low noise performance, is used to construct the voltage follower. 

**Figure 8:**
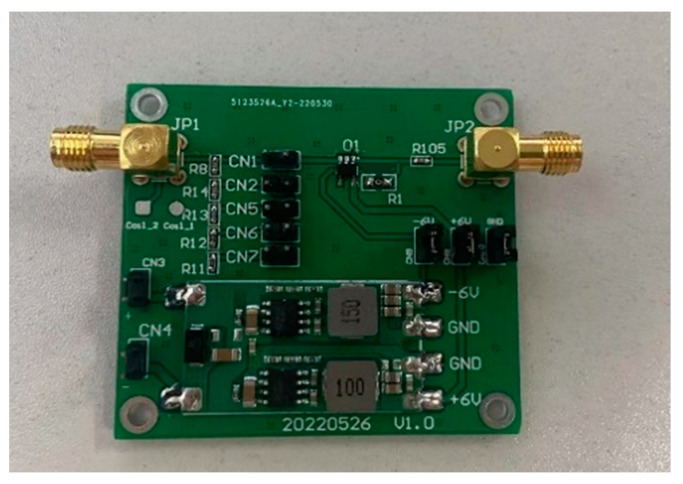
Multi-range PCB board with impedance matching function.

We designed the voltage divider resistor according to Equation (17). As the frequency bands of the lightning currents, current components A and H, are quite different, this leads to a large difference in the amplitude of the coil-induced voltage. From Equation (17), it can be inferred that, for the magnetic field generated by lightning current component A, the induced voltage generated by 10 coils is between 34 mV and 34 V when the magnetic field amplitude varies from 5 A/m to 5000 A/m; whereas for component H, when the magnetic field amplitude is 5 A/m to 1000 A/m, the induced voltage generated by 10 coils is between 970 mV and 195 V. Taking the large difference in coil-induced voltage into consideration, the voltage divider ratio is, therefore, set to 0.1, 0.01, and 1 time. The parameters of the voltage divider are shown in Table 1. Among these parameters, the 2.2 kΩ resistor is connected in series with three 680 Ω resistors, and the finished Printed Circuit Board (PCB) board is shown in Figure 8.

### 4.2. Error Analysis 

The measurement error of the sensor mainly comes from the calibration and the B-ssdot itself, for example, stray parameters, sampling resistances, signal reconstruction, etc. On account of this, this section is organized to analyze the measurement error of the sensor.

#### 4.2.1. The Calibration System Error

In order to investigate the measurement error of the calibration system, we utilize COMSOL simulation software to simulate the calibration system, then carry out the calibration experiments.

**Figure 9:**
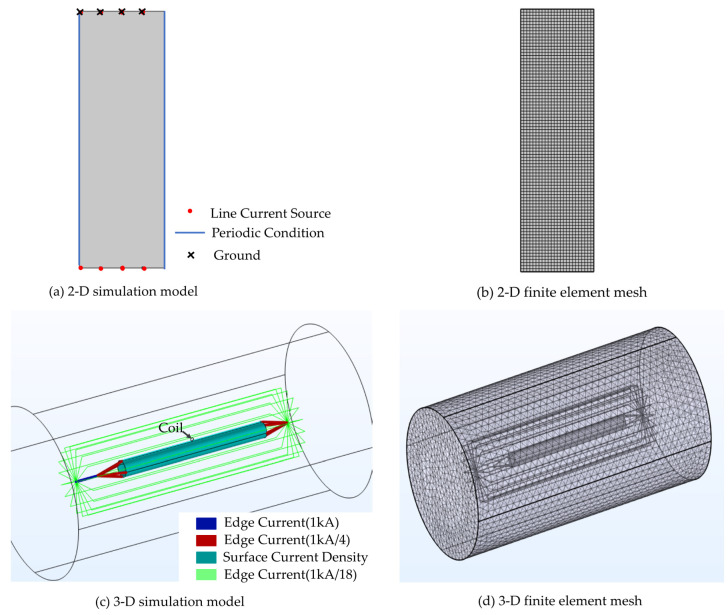
Numerical model of calibration system including excitation settings for 2-D model (**a**), and 3-D model (**c**), mesh setup for 2-D model (**b**), and 3-D model (**d**).

The whole numerical model and mesh are shown in Figure 9. We use the current and magnetic field module in COMSOL Multiphysics 6.0. To improve the convergence of the model, the surface current density of the aluminum barrel under steady-state conditions is first determined by using the 2-D model. In the 2-D model, the line current source is set to 1 A. In the meshing of the 2-D model, set the maximum element size to 20 mm, which is much less than one-tenth of the wavelength. We first specify the short side as the distribution and set the number of cells to 6. The entire domain is meshed by mapping the mesh. The total number of elements is 2040 with 3,320,425 degrees of freedom.

In the 3-D numerical model, a coaxial structure is formed by an aluminum barrel and wires. The radius of the aluminum barrel is 90 mm and the barrel length is 1700 mm. In the 3-D model, we set the maximum element size to 100 mm. To project the current density of the 2-D aluminum barrel surface to the 3-D aluminum barrel surface, the top round edge of the aluminum barrel must also be set as a distribution, and the number of fixed units is 6, which is consistent with the 2-D model. Because the coil is smaller relative to the aluminum barrel, a separate meshing of the coil is required. Mesh refinement of the coils is achieved by sweeping and distributing. The total number of degrees of freedom is 3,346,993. The return circuit is composed of copper wires. To ensure the uniformity of the magnetic field distribution, the return conductors (18 copper wires) are evenly distributed around the aluminum barrel, and are replaced by edge currents. The distance between the return conductor and the aluminum barrel is 285 mm. The injected lightning current is a 1 kA lightning current component H. The surface current density in the 3-D environment is then obtained by using the transformation relationship between the cylindrical coordinate system and the Cartesian coordinate system. The line current density on the return conductor is set according to Kirchhoff’s current law, and the specific setting is shown in Figure 8. Finally, the induced voltage on the coil is obtained by building a physical coil. 

**Figure 10:**
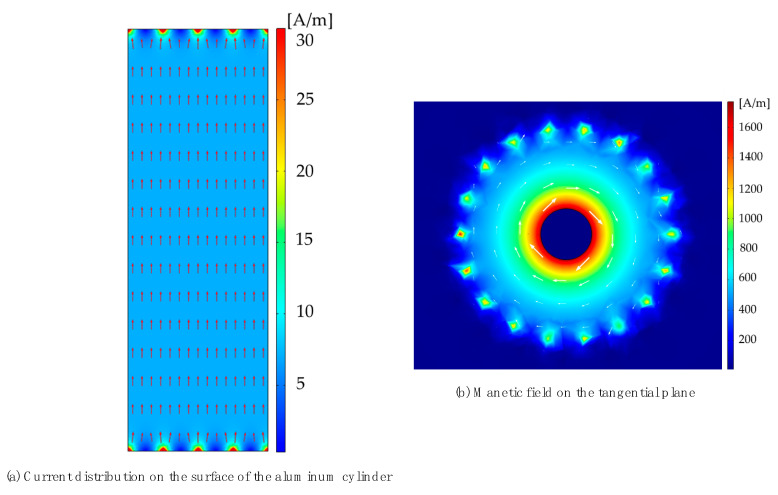
The surface current (2-D model) and magnetic field on the tangential plane of the aluminum barrel (3-D model).

The current density after the surface of the aluminum cylinder is unfolded and the magnetic field (245 ns, 4 μs) on the tangential plane of the aluminum barrel are shown in Figure 10.

Figure 10 reveals that the current density is larger at the injection point and the outflow point, and is relatively uniform at the middle of the aluminum barrel. In the tangential plane, the magnetic field decreases away from the aluminum barrel, but this is not enough to analyze the inhomogeneity of the magnetic field, which needs to be quantitatively described by a formula. The D fluctuation on the calibration. The non-uniformity could be evaluated by

(19) $$\delta =\frac{J_{\max}-J_{\min}}{0.5\times \left(J_{\max}+J_{\min}\right)}\times 100\%,$$

where J represents the current density, and max and min in the following table represents the maximum value and the minimum value, respectively. 

To calculate the non-uniformity in the calibration environment, the magnetic field detection ring is 27 mm away from the aluminum cylinder at the wavefront and half-peak times, respectively. According to Equation (19), the non-uniformity of the surface current of the aluminum barrel at the peak time and half-peak time is calculated to be 1.5% and 1.4%, respectively.

The inhomogeneity of the magnetic field is less than 1.5%, and the fluctuation of the magnetic field is small. In the calibration environment proposed in this paper, the magnetic field fluctuation caused by the influence of the wire can be ignored.

After investigating the inhomogeneity of the magnetic field, the error mainly comes from the installation height of the sensor, the rotation angle, and the non-uniformity of the sensing area.

We consider the inhomogeneity of the magnetic field in the magnetic induction region and the error when installing the coil. Therefore, the changes in the induced voltage in the coil were simulated when the sensor was 10 mm, 15 mm, and 20 mm away from the aluminum cylinder, and rotated by 5°, 10°, and 20° to investigate the measurement error.

The induced voltage and its deviation in the coil, according to the simulation results, are shown in Table 2. The error of the Equation (15) calculating method and the traditional calibration method are also compared. The traditional calculation equation ignores the inhomogeneity of the magnetic field in the induction area and uses the magnetic field H = I/2 πr at the center of the coil to represent the magnetic field in the entire induction area.

According to Table 2, it can be seen that, if the non-uniformity of the induction area is not considered, the induced voltage of the coil will be very large, and the deviation can reach 20% in this calibration environment. This can seriously reduce the accuracy of the sensor. Therefore, our proposed calibration formula can effectively reduce the error.

In addition, the sensitivity of the sensor was also calibrated. The coaxial calibration system is the same with the simulation configuration. In this test, the capacitance of the capacitor in the generator is 0.4 μF, the wave-modulating resistance is 12 Ω, the wave-modulating inductance is not connected, the loop inductance generates approximately 0.65 μH, and the charging voltage of the capacitor is adjustable from 0–80 kV. Pearson model 1423 and Pearson model 411 transformers are used to measure lightning current component A and the component H wave, respectively. The outer conductor of the coaxial structure is grounded, the inner conductor is connected to a high-voltage terminal, and the sensor is placed in the middle of the aluminum cylinder, which is connected with the voltage follower and the oscilloscope by a coaxial cable (RG58).

**Figure 11:**
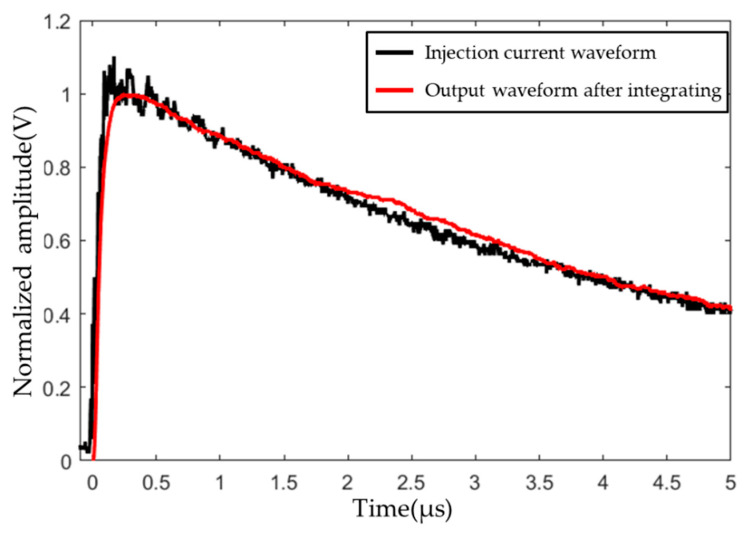
Comparison of the lightning current component H injection (black line) and integrated waveforms (red line 1).

Figure 11 shows the comparison between the integrated waveform and the injected waveform of the sensor measurement model.

**Figure 12:**
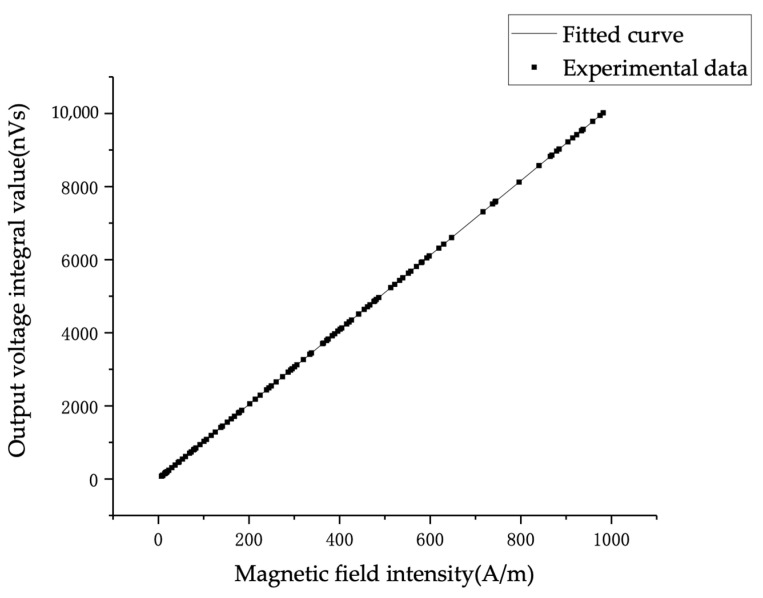
The relationship between the amplitude of the output signal after integrating in the calibration environment and the injected current.

The sensor is calibrated, the capacitor charging voltage range is from 0 V to 80 kV, 150 discharges are performed, and the proportional coefficient of the sensor is 0.98 × 10^8^. The calibration curve is shown in Figure 12.

**Figure 13:**
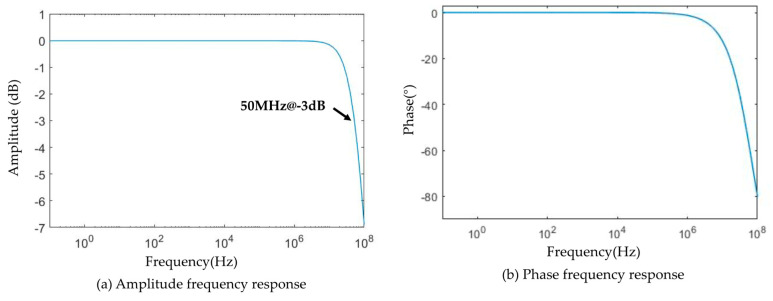
The high-frequency cut-off frequency of the sensor is 50 MHz.

For the bandwidth of the sensor, the amplitude-frequency response curve of the sensor can be obtained according to the measured values of the stray parameters of the coil, as shown in Figure 13. The −3 dB cut-off frequency is at 50 MHz, which is much higher than the frequency band of the lightning current waveform. This demonstrates that the B-dot sensor designed in this study could totally cover the lightning frequency. The frequency band of the voltage follower constructed by OPA659 can reach 650 MHz at unity gain, which is not the main factor limiting the frequency band of the sensor.

The error caused by the transmission process of the coaxial line should also be analyzed because the magnetic field coil is connected to the oscilloscope after connecting the voltage follower through the coaxial line. 

**Figure 14:**
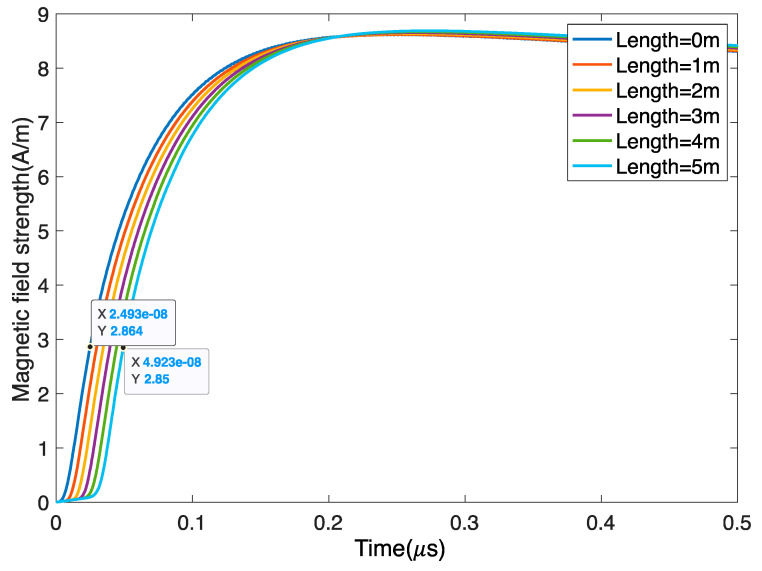
Influence of the coaxial cable length on the signal. With an increase in the length, the signal gradually becomes slower and the amplitude decreases, but the influence is small.

We discover that the spurious parameter of the RG58 coaxial line is 100 pF/m, and then use the 10-level LC link to be equivalent. Next, the output signal is numerically integrated by using the trapezoidal method. The length of the coaxial line changes from 0 m to 5 m, and the output waveform after integration is shown in Figure 14. For the RG58 coaxial cable, due to the delay in the transmission line, the rising edge of the waveform is delayed by approximately 5 ns for each additional 1 m, and the amplitude decreases by 0.016 A/m after integration. Therefore, part of the measurement error can be eliminated by multiplying by a factor or by using a coaxial cable of the same length.

#### 4.2.2. The Error of the B-Dot and the Circuit

After investigating the measurement error of the calibration system, this section analyzes the error of the sensor and the circuit by calculating and testing.

For the skin effect, the calculation results of Equations (5)–(7) are verified by using finite element software. Table 3 shows the calculated and measured results of the spurious parameters.

The stray inductance, stray capacitance, and coil internal resistance determined by using software are approximately 8.47 μH, 0.249 pF, and 2.4 Ω, respectively. By using the TH2821B bridge to measure the inductance, stray capacitance, and resistance, the results are 7.9 μH, 0.2 pF, and 2.2 Ω, respectively. The theoretically calculated stray inductance, stray capacitance, and coil internal resistance are equal to 6.69 uH, 0.216 pF, and 2.38 Ω, respectively. According to the measured and simulated data, it can be seen that the error of the theoretical calculation formula is very small.

**Figure 15:**
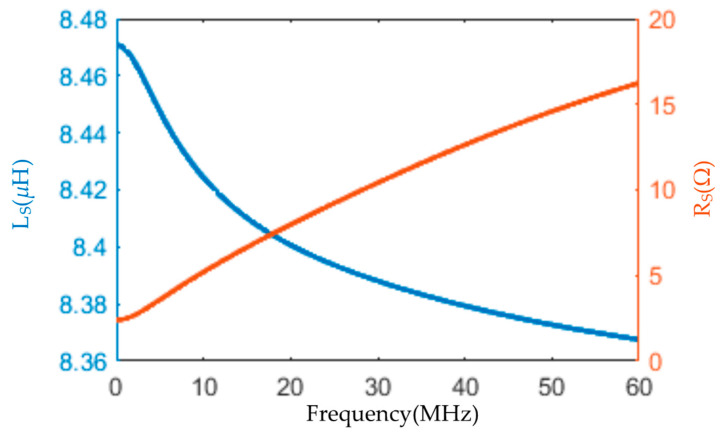
The change in inductance with frequency is small, and the resistance increases gradually with the increase in frequency.

To analyze the influence of the skin effect on the internal resistance of the coil, the curves of the coil resistance and inductance are obtained by using the frequency sweep function, as shown in Figure 15.

The resistance, as presented in Figure 15 (red line), increases gradually with the frequency due to the skin effect. The coil resistance is 2.4 Ω at low frequency and 5 Ω at 10 MHz. At 10 MHz, less than 0.1% of the influence on the input voltage of the op-amp is caused by the resistance. Therefore, the influence of the error caused by the skin effect can be ignored. 

Figure 15 (blue line) is the change in coil inductance with the frequency varying from 0 to 60 MHz. From the figure, it can be inferred that it is reduced by approximately 0.1 uH. Rs + ωL << R_L_ within the frequency band of the high-frequency lightning current signal can ensure that the coil works in a differential mode.

To determine the actual divider ratio and the high-frequency capability of the divider resistors, the divider ratio is calibrated with a signal generator and an oscilloscope. The signal generator used is Keysight’s 33600A Series waveform generator, and the oscilloscope is MSO9404A from Agilent Technologies. The power supply of the op-amp is QJ3005H, and the PCB board is equipped with an additional voltage regulator unit to ensure the stability of actual use. The signal generator is connected to the input of the voltage follower, and the output of the voltage follower is connected to the oscilloscope. According to the test, the actual voltage divider ratio is 0.009, 0.1, and 1.067.

The error sizes of the different trend item removal methods are compared, and 4, 40, and 60 samples are randomly selected for the calibration data of 1.067, 0.1, and 0.009 voltage divider ratios, respectively. The maximum relative error of all samples is shown in Table 4. Method A uses the nonlinear least squares method to reconstruct the current waveform, and method B uses the mean value when the signal is 0 to remove the trend term. It can be seen from Table 4 that the trend item removal method proposed in this paper can effectively identify the half-peak time, and the maximum peak error is 3.1% of the full scale. The wavefront time is shorter than the half-peak time, and the error accumulation is small. Although the traditional method can effectively read the wavefront time, it is, however, difficult to identify the falling edge of the waveform with the accumulation of errors.

### 4.3. Application

**Figure 16:**
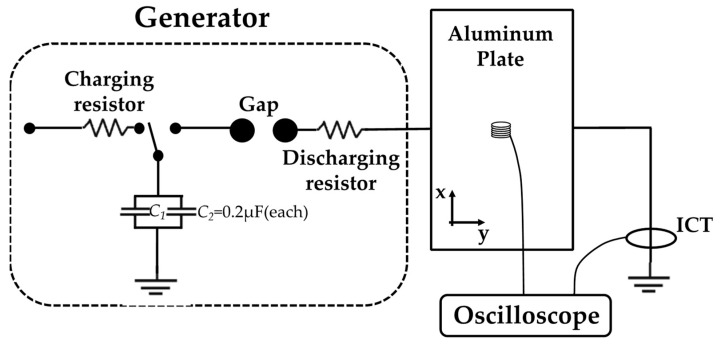
The C520 and B-dot experiment measuring the surface current distribution and magnetic field strength of the aluminum plate.

We measured the surface current of the aluminum plate using B-dot. The aluminum plate is the main material for the connection of capacitors, wave modulating resistors, and wave modulating inductors in pulse current generators. It is very important to analyze the current distribution on the surface of typical plates. In addition, in order to verify the performance of the proposed sensor, the current distribution on the surface of the plate was compared and measured. The experimental principle is shown in Figure 16. An aluminum plate with a length of 1000 mm, a width of 660 mm, and a thickness of 1 mm was chosen. The midpoint of the long side is the current injection end. In addition, a 2-D coordinate system on the slab layer was established, with a cell size of 100 mm × 100 mm. The lower left corner was set as (0, 0), and the geometric center of the plate was marked (500, 330).

**Figure 17:**
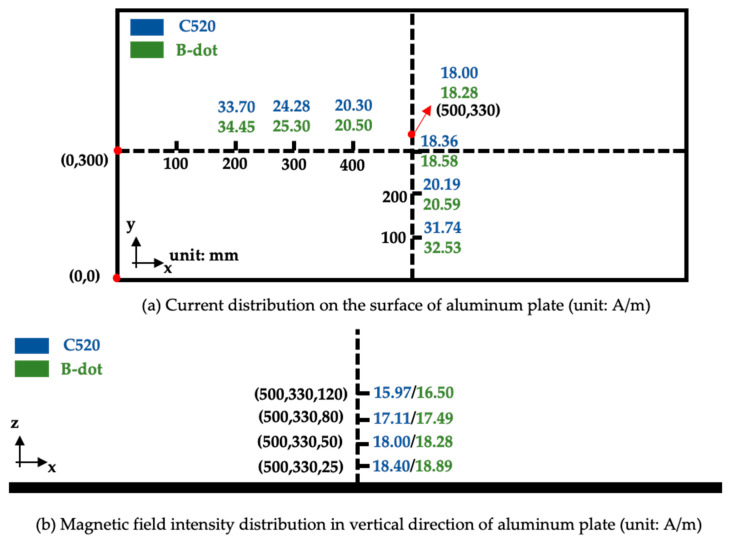
Comparison of C520 and B-dot in measuring the surface current distribution and magnetic field strength of the aluminum plate.

The sensor measuring the current is a Pearson model 411. The waveform generator is the same as the one used during calibration, and the lightning current component H wave is used for injection. By comparing the magnetic field sensor with model ASM-1M (serial number C520, Coliy Company), the measurement error of our proposed sensor is analyzed. The C520 is calibrated first, and the sensor output ratio is 1.175 (mG/mV) after calibration. The final measured surface current of the plate is shown in Figure 17.

According to the measurement results, whether it is the long side or the short side of the aluminum plate, the surface current of the plate is larger as it is closer to the edge, as a result of the skin effect. The surface current increases faster along the short axis than the long axis. The maximum error between the calibrated C520 and the sensor proposed in this paper is 1.02 A/m when measuring the surface current.

## 5. Conclusions

In this paper, a multi-range surface current sensor was proposed based on Maxwell’s equations, and a voltage follower was used to match the input resistance of the oscilloscope. We optimized the oscillation problem of the output signal and provided the value range of the sampling resistor. A low-cost and efficient calibration method was proposed, and an accurate calibration formula was given. The noise-free signal was reconstructed by using the nonlinear least squares method. Finally, the error of the sensor was analyzed in detail.

The error of the calibration system mainly comes from the inhomogeneity of the normal and tangential magnetic fields of the aluminum barrel. According to the simulation, the inhomogeneity of the tangential magnetic field is within 1.5%. The influence of the non-uniformity of the tangential magnetic field can be removed by the calibration formula provided. The linearity of the sensor was calibrated for multiple ranges and its bandwidth was analyzed. For the error of the sensor itself, the influence of the skin effect and the error of the waveform reconstruction algorithm were mainly studied. The research in this paper greatly improves the accuracy of the surface current sensor to measure the high-frequency lightning current component.

## Figures and Tables

**Table 1:** Voltage divider resistor value.

Current Type	Magnetic Field Amplitude (A/m)	Change Rate of Magnetic Field	Induced Voltage (V)	Voltage Divider Ratio	Op Amp Input Voltage (V)	Divider Resistor Value (Ω)
A	0–300	2.1 × 10^8^	2.055	1	2.055	2.2 k
	300–3 k	2.1 × 10^9^	20	0.1	2	220
	3 k–30 k	2.1 × 10^10^	205	0.01	2.05	20
H	0–10	2 × 10^8^	1.9	1	1.9	2.2 k
	10–100	2 × 10^9^	19	0.1	1.9	220
	100–1000	2 × 10^10^	195.87	0.01	1.9587	20

**Table 2:** Comparison of calculation formula and simulation.

Deviation of the Coil	The Induced Voltage of the Coil		
	Simulation	Theoretical Calculation	Traditional Calculation
10 mm from the tube 15 mm from the tube 20 mm from the tube	228.9	229.4	310
	228.7	229.3	297
	228.4	229.2	285
Rotation angle 5° Rotation angle 10°	227.9	228.5	309
	226.3	225.9	305
Rotation angle 20°	215.9	215.5	291

**Table 3:** Results of spurious parameters.

	L_S_ (μH)	C_S_ (pF)	R_S_ (Ω)
Experiment	7.9	0.2	2.2
Analytical formula	6.69	0.216	2.38
Simulation	8.47	0.249	2.4

**Table 4:** Comparison of trend item removal methods.

Methd	A	B
Peak value error	3.1%	7.4%
Wavefront time error	2.8%	6.9%
Half-peak time error	3.7%	not recognized

## Data Availability

Not applicable.
